# Return to Work in Patients With Unilateral Inguinal Hernia Surgery: A Comparative Study Between Laparoscopic Transabdominal Preperitoneal Approach and Lichtenstein Tension-Free Mesh Repair

**DOI:** 10.7759/cureus.39202

**Published:** 2023-05-18

**Authors:** Abdul Hakeem, Sabah Uddin Saqib, Hasnain Zafar

**Affiliations:** 1 Department of Surgery, Aga Khan University Hospital, Karachi, PAK

**Keywords:** tension-free mesh repair, laparoscopic hernia repair, return to work, tapp, lichtenstein repair, mesh repair, inguinal hernia

## Abstract

Objective

The objective of this prospective cohort study was to compare the time to return to work between patients who underwent laparoscopic transabdominal preperitoneal (TAPP) hernia repair and those who underwent Lichtenstein tension-free hernia repair with mesh for unilateral inguinal hernia.

Methodology

Patients were registered for unilateral inguinal hernia review at Aga Khan University Hospital, Karachi, Pakistan, from May 2016 to April 2017 and followed till April 2020. All patients aged 16-65 planned for unilateral transabdominal preperitoneal hernia repair or Lichtenstein tension-free hernia mesh repair were included. Patients with bilateral inguinal hernia repair, limited activity, or above retirement age were excluded. A non-probability consecutive sampling technique was implemented, and patients were divided into two cohort groups: Group A underwent laparoscopic transabdominal preperitoneal hernia repair, while Group B underwent Lichtenstein tension-free mesh repair. Patients were followed up at one week to inquire about the resumption of activities and then at one and three years for recurrence.

Results

Sixty-four patients met the inclusion criteria; three patients opted out of research, and 61 patients agreed to participate; one patient was excluded due to the conversion of the procedure. The remaining 30 in Group A and 30 in Group B were followed for the study period. The mean time to return to work in Group A was 5.33 ± 4.46 days; in Group B, it was 6.83 ± 4.58 days, with a p-value of 0.657. One recurrence was observed at three years in Group A.

Conclusion

Although the time to return to work at our hospital was slightly shorter in laparoscopic hernia repair than in the open technique, the results were not statistically significant. In addition, there was no significant difference in hernia recurrence at the one-year follow-up between laparoscopic transabdominal preperitoneal hernia repair and Lichtenstein tension-free hernia mesh repair for unilateral inguinal hernia.

## Introduction

Inguinal hernia repair is one of the most commonly performed general surgical procedures worldwide. Traditional open surgical repair (Lichtenstein) has been widely replaced by laparoscopic hernia repair in many parts of the world due to its fast recovery and early return to work [1]. Laparoscopic hernia repair has been shown to have a similar incidence of recurrence compared to the Lichtenstein technique [2]. However, there are still some discrepancies in the standardization of the laparoscopic hernia repair procedure, especially in cases of unilateral hernias, due to the diversity of factors such as cost, availability of instruments, expertise, type of hernia, and laterality [3].

In contrast to laparoscopic hernia repair, the Lichtenstein hernia repair can be performed as daycare surgery under local anesthesia [2,4-6]. Although laparoscopic hernia repair is safe and effective, it requires a longer learning curve with more complications during the learning phase [3]. The benefits can only be attributed to countries with sufficient resources [3,7]. In Pakistan, where resources are limited, Lichtenstein hernia repair is a routine surgical procedure due to a lack of expertise and finances that patients have to bear. In such countries, laparoscopic hernia repair costs are relatively high compared to the average income per capita of male employees [8], the primary breadwinners in most households.

Moreover, patients who undergo surgical procedures tend to resume their work late, resulting in a loss of household resources. Therefore, a surgical procedure that enables patients to resume their activities at the earliest should be performed. We believe the patient can compensate partially or fully for the cost of surgery if he returns to work early. In addition, recurrence can add up to the cost of hernia repair; most studies have shown comparable recurrence rates between transabdominal preperitoneal (TAPP) and Lichtenstein hernia repair [3].

Our study aims to compare the return to work and rate of recurrence in patients who undergo TAPP hernia repair with those who undergo Lichtenstein hernia repair. This study will be instrumental in improving patients' quality of life undergoing hernia repair by providing them with the best possible surgical procedure that meets their needs.

## Materials and methods

This prospective study aimed to compare the return to work of patients aged 16 to 65 years who underwent TAPP versus Lichtenstein hernia repair with unilateral inguinal hernia repair and the recurrence rates. The study was conducted at Aga Khan University Hospital between May 1, 2016, and April 30, 2017, with follow-up visits at one and three years, concluding in April 2020.

The sample size calculation was performed using OpenEpi version 3.01 software, which considered the median return to work of 21 days for open surgery and 14 days for laparoscopic surgery [9]. The study was designed with a two-tailed test, a significance level of 0.05, a power of 0.90, and a 95% confidence interval. The calculated sample size for each group was 20. However, to account for potential loss to follow-up, the sample size was increased to 30 participants per group. The sample size calculation was based on the assumption that the distribution of the outcome variable is normal and the effect size is the difference between the medians of the two groups. However, it is important to note that other assumptions, such as the homogeneity of variance, were not tested. Nevertheless, we believe that the calculated sample size provides sufficient power to detect a clinically meaningful difference in return to work between open surgery and laparoscopic surgery.

We employed a non-probability consecutive sampling technique to select patients who had scheduled unilateral inguinal hernia repair appointments. All patients were provided with comprehensive information about laparoscopic transabdominal preperitoneal and Lichtenstein tension-free hernia repair techniques and their potential complications. Patients were given complete autonomy in choosing their preferred surgical approach without any influence from the researchers. Patients with appointments specifically with a consultant performing totally extraperitoneal (TEP) repair were excluded, as this technique was not routinely performed at our hospital. Those who declined to participate in the study were also excluded. Ultimately, we enrolled 30 consecutive patients who underwent laparoscopic transabdominal preperitoneal repair (Group A) and 30 who underwent Lichtenstein mesh repair (Group B) for unilateral inguinal hernia. One patient in Group A required conversion from laparoscopic to open repair and was excluded from the study. An additional patient was included in Group A to balance the numbers.

All surgeries were performed by experienced surgeons. Postoperatively, patients were prescribed Nuberol Forte (650 mg/50 mg paracetamol/orphenadrine) and Rotec (diclofenac/misoprostol 50 mg/0.2 mg) in both groups at regular intervals. Patients were encouraged to resume their activities as soon as possible, and one week after surgery, each patient was contacted for inquiries about their activities and level of comfort. Return to work was divided into outdoor, office sitting, and household work. The level of comfort was categorized into subjective categories: quite comfortable, mild discomfort with no limitation to activity, pain limiting regular work, and pain limiting to bed. The financial class was divided into non-insured or insured. Demographic data collected included age, gender, and body mass index (BMI). Patients were followed up at one and three years for the recurrence. Those who underwent significant changes in surgeries, such as conversion to open repair, repair of bilateral hernia, TEP repair, or additional procedures, were excluded from the research.

The two groups were compared for their demographics, financial status, type of work, level of comfort, and return to activity. Data were analyzed using Minitab version 21.5 for Windows, and the chi-square test was used. A p-value of less than 0.05 was considered significant. In addition, Classification and Regression Tree (CART) analysis was conducted to correlate the level of comfort.

This study was registered with the Ethical Review Committee of Aga Khan University Hospital, with registration number 3583-Sur-ERC-15.

## Results

After applying the exclusion criteria, 60 patients (30 in each group) were enrolled for analysis out of the initial 64 patients. Three patients dropped out of the study (two in Group A and one in Group B), and one patient underwent a conversion of the procedure; we excluded these patients from the analysis. All patients were males; the mean age in Group A was 47.8 (±10.54) years, and in Group B, it was 53.7 (±13.92) years. The mean body mass index of patients in Group A was 25.99 (±3.71) kg/m^2^, while in Group B, it was 23.53 (±3.69) kg/m^2^. The demographics of patients in each group are summarized in Table 1.

**Table 1:** Demographics of patients in each group SD: standard deviation, BMI: body mass index

	Group A	Group B
Numbers	30	30
Mean age (SD)	47.8 (±10.54)	53.7 (±13.92)
Gender		
Male	30	30
Female	0	0
BMI	25.99 (±3.71)	23.53 (±3.69)

Table 2 shows that in Group A, 20% of patients were involved in household activities, 66.7% had office jobs, and 13.3% had outdoor activities. In contrast, 46.7% of patients in Group B were involved in household activities, 40% in office work, and 13.3% in outdoor activities. In addition, all 30 patients in Group A were self-paid; in Group B, 28 (93.3%) patients were non-insured, and two (6.7%) patients had insurance coverage for surgery.

**Table 2:** Nature of work and financial coverage of patients for hernia repair in both groups

	Group A	Group B
Nature of working activities		
Household	6 (20%)	14 (46.7%)
Office sitting	20 (66.7%)	12 (40%)
Outdoor	4 (13.3%)	4 (13.3%)
Financial coverage of surgery		
Non-insured	30 (100%)	28 (93.3%)
Insured	0	2 (6.7%)

The mean duration of return to work in Group A was 5.33 ± 4.46 days; in Group B, it was 6.83 ± 4.58 days, with no statistically significant difference (p-value = 0.657, post hoc power = 25%). No hernia recurrence was noted in the laparoscopic or Lichtenstein technique group at a follow-up period of one year. Still, at three years, there was one recurrence in the laparoscopic group for unilateral inguinal hernia repair, which was subsequently repaired as an open technique. Table 3 summarizes the days required to return to work in each group and the recurrence rate.

**Table 3:** Number of days required for return to work in each group and the rate of recurrence

	Group A	Group B	p-value (post hoc power)
Return to work	5.33 ± 4.46	6.83 ± 4.58	0.657 (25%)
Recurrence at one year	0	0	
Recurrence at three years	1	0	

**Figure 1:**
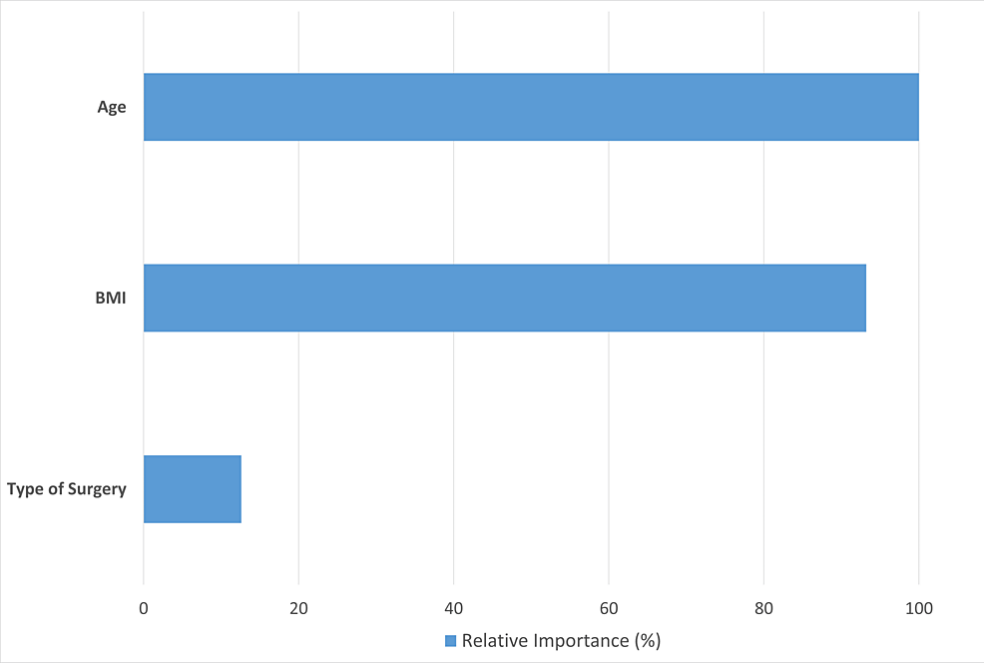
CART analysis: level of importance (relative variable importance) Variable importance measures model improvement when splits are made on a predictor. Relative importance is defined as % improvement with respect to the top predictor. CART: Classification and Regression Tree, BMI: body mass index

The CART analysis revealed that among the variables investigated, the type of surgery had a relative importance of 12% in predicting the level of comfort and return to work. In contrast, age had a relative importance of 100%, and BMI had a relative importance of 93.2%. These findings suggest that age and BMI are more important predictors of comfort and return to work than the type of surgery. Furthermore, the receiver operating characteristic (ROC) curve prediction was greater than 0.9 for all areas of comfort, indicating a high accuracy in predicting the level of comfort based on the variables investigated in our study, as shown in Figure 1 and Table 4.

**Table 4:** CART analysis summary and ROC for level of comfort CART: Classification and Regression Tree, ROC: receiver operating characteristic

Total predictors	3
Important predictors	3
Number of terminal nodes	10
Minimum terminal node size	3
Misclassification cost	0.0705
Area under the ROC curve	
Level of comfort = discomfort limiting regular work versus not	0.9746
Level of comfort = discomfort limiting to bed versus not	0.9831
Level of comfort = mild discomfort with no limitation to activity versus not	0.9537
Level of comfort = quite comfortable versus not	0.9243

## Discussion

Over the past few years, numerous studies have compared the outcomes of laparoscopic and open hernia repair surgeries worldwide, including in Pakistan. The primary goal has been to identify the benefits of each approach.

Most well-conducted randomized controlled trials (RCTs) have demonstrated that laparoscopic surgery has lower recurrence rates than open surgeries in the long term [3,7]. However, postoperative pain and recovery are almost similar for both approaches. A significant difference in bilateral hernia repair favors laparoscopic over open hernia repair. In cases of recurrent hernia, postoperative recovery and long-term follow-up are better in laparoscopic surgery [2,9-23].

Two studies conducted in 2016 by Latif Bahram [12] and Akhtar et al. [24] compared laparoscopic and open hernia repair surgeries and concluded that laparoscopic surgery resulted in an early return to work for patients. Latif Bahram [12] compared 150 TAPP and 150 Lichtenstein repair (LR) cases and found that patients who underwent TAPP repair returned to work earlier than those who underwent LR. Similarly, Akhtar et al. [24] conducted an RCT comprising 50 LR and 30 TAPP cases and concluded that patients who underwent TAPP repair returned to work earlier than those who underwent LR. Both studies showed a significant difference in the time patients return to work after surgery.

Two RCTs evaluated the early return to work and quality of life following laparoscopic and open hernia repair and found no significant difference between the two techniques. Dhankhar et al. (2014) [25] observed almost no difference in the quality of life between the two procedures in their RCT, which included 30 Lichtenstein and 29 TEP patients. Similarly, Langeveld et al. (2010) [13] reported a significant difference of two days in return to work between the TEP and Lichtenstein groups (p = 0.001), but no significant difference in the quality of life (p = 0.35). These findings suggest that both laparoscopic and open hernia repair techniques can result in similar outcomes regarding early return to work and quality of life.

In contrast, Salma et al. (2015) [26] compared the postoperative pain and length of stay of patients who underwent laparoscopic and open hernia repair surgeries. The study included 30 LR and 30 TAPP cases and found that postoperative pain was significantly higher in LR compared to the TAPP approach. However, this study did not evaluate the time taken for patients to return to work after surgery. Postoperative pain and the need to return to work quickly are significant concerns after inguinal hernia repair. Non-opioid analgesics, such as parecoxib and acetaminophen, are effective and have minimal side effects and cost. Combining IV acetaminophen with either intramuscular pethidine or IV parecoxib is superior to IV acetaminophen alone for pain control in patients undergoing open inguinal hernia repair [27].

The choice of hernia repair approach depends on the patient's clinical condition, the surgeon's experience, and the cost-effectiveness of the surgery. In low- to middle-income countries such as Pakistan, where health insurance is not typical, the cost-effectiveness of surgery is also a significant factor. Open hernia repair is still more cost-effective for unilateral inguinal hernia. Given the sole financial supporter in most families in Pakistan, the number of days lost to work is a significant determinant. Therefore, short-term outcomes, such as when patients return to work, should also be given high importance. However, laparoscopic surgery should be considered for bilateral hernia repair and recurrent hernia cases, resulting in better postoperative recovery and long-term benefits.

The study has some notable limitations that should be considered when interpreting the results. These limitations include the relatively small sample size, which may have limited the study's statistical power to detect significant differences between the two groups' early return to work. Moreover, the study's follow-up period was limited to three years, which may not have been sufficient to detect long-term complications and recurrences. Additionally, the study did not assess postoperative pain using a visual analog scale or quality of life, which are important factors to consider when evaluating the benefits of different hernia repair techniques. Lastly, the non-randomized nature of the study may have introduced bias and confounding factors that could affect the results. Future studies, particularly well-controlled randomized trials with propensity score matching, are recommended to overcome these limitations and provide more robust evidence for the benefits of different hernia repair techniques.

## Conclusions

Our study found no significant difference in return to work time or recurrence rates between laparoscopic transabdominal preperitoneal repair (TAPP) and Lichtenstein mesh repair for unilateral inguinal hernia in patients aged 16-65. Age and BMI were more important predictors of comfort and return to work than the type of surgery. The choice of hernia repair approach should be based on the patient's clinical condition, surgeon experience, and cost-effectiveness of the surgery, especially in low- to middle-income countries such as Pakistan.
